# Musculoskeletal health in children and adolescents

**DOI:** 10.3389/fped.2023.1226524

**Published:** 2023-12-15

**Authors:** Maria Felicia Faienza, Flavia Urbano, Mariangela Chiarito, Giuseppe Lassandro, Paola Giordano

**Affiliations:** ^1^ Pediatric Unit, Department of Precision and Regenerative Medicine and Ionian Area, University of Bari “Aldo Moro”, Bari, Italy; ^2^ Giovanni XXIII Pediatric Hospital, Bari, Italy; ^3^ Department of Interdisciplinary Medicine, University of Bari “Aldo Moro”, Bari, Italy

**Keywords:** musculoskeletal health, children, adolescents, irisin, physical activity, sports, rare diseases

## Abstract

The purpose of this narrative review was to investigate the key determinants of musculoskeletal health in childhood and adolescence, with particular attention to the role of physical activity. First, we examined the importance of bone modeling and remodeling in maintaining the bone health and the integrity and mechanical characteristic of the skeleton. In addition, we reported the evidence on an appropriate calcium and vitamin D intake, as well as local load variation in achieving proper peak bone mass. Proteomic and transcriptomic studies identified the skeletal muscle “secretoma”, consisting of several myokines involved in endocrine and paracrine functions. Among these, we explored the role of irisin, a myokine involved in the muscle-bone crosstalk, and in the regulation of metabolic pathways. It is known that physical activity during growing positively impacts on skeleton and can protect by bone loss in adulthood. However, there are still concerns about the optimal interval duration and exercise intensity, particularly at the pubertal growth spurt which represents a window of opportunity to increase skeletal strength. We reported data from clinical trials performed in the last 5 years analyzing the impact of the type and timing of physical activity during childhood on skeletal development. Finally, we reported recent data on the significance of physical activity in some rare diseases.

## Introduction

The coordinated action of osteoblasts, osteoclasts and osteocytes directs bone modelling and remodeling (1). The former process promotes longitudinal growth of the bones and adaption of the skeleton to mechanical stress, while the latter removes old or damaged bone. Thus, bone remodeling maintains the strength of the skeleton over the course of life. During bone modeling, osteoclasts and osteoblasts work individually, while when bone remodeling occurs, bone resorption and bone formation are coupled into bone remodeling units (1). With ageing, the skeleton undergoes substantial architectural and metabolic modifications, possibly predisposing to osteoporosis and increased risk of fractures. Although osteoporosis is typically associated with ageing, the contributing factors can act already during growth. Hence, the impairment of bone health occurs during the developmental age, when over a third of bone mass is accumulated, reaching a peak around the second decade of life (2). The conjunction of this critical period of bone growing with bone loading and physical activity represents a “window of opportunity” to develop a healthy skeleton (3). Environmental factors, such as diet and exercise, impact 20%–40% of peak bone mass in adulthood. Physical activity is recognized as a means of health promotion and disease prevention throughout the life (4), although there are few specific recommendations in infancy and childhood, which represent periods of life wherein healthy behaviors can have lasting metabolic and behavioral consequences (5, 6). An adequate development of skeletal muscles during these two periods may have long-term consequences on body composition and inclination to engage in physical activity throughout life (7). Moreover, although muscle fiber composition is genetically determined (8), early physical training can play an important role in “fiber reprogramming” (9). Bone mineral content (BMC) and bone mineral density (BMD) increase in response to repetitive and variable loading activities through increased force and strain. Moderate or intense physical activity and several sports inducing greater than normal bone load are critical for the achievement of bone strength (10). Considering this, the implementation of exercise interventions during childhood and adolescence could maximize peak bone mass and consequently slow down the onset of osteoporosis.

In this narrative review, we focused on the key determinants of musculoskeletal health in pediatric age, and we reported the most recent studies on the impact of physical activity on bone strength during childhood and adolescence. Furthermore, we reported the effects of physical activity in some rare diseases in which has been demonstrated an improving of bone health.

## The concept of bone health: bone modeling, bone remodeling and peak bone mass

Bone contains an organic component represented by collagenous and non-collagenous proteins and cells, and a mineral part of hydroxyapatite (11).

Bone cells include osteoblasts, the bone forming cells, which originate from mesenchymal stem cells; osteocytes, differentiated from osteoblasts and inserted in the bone matrix; and osteoclasts, the bone reabsorbing cells, differentiated from hematopoietic progenitors. Skeletal growth is the result of the bone expansion of cortical bone, and bone growing through endochondral ossification (12). This process called “bone modeling”, which starts during fetal life and proceeds until epiphyseal fusion, is particularly sensitive to mechanical load, supporting the significance of physical activity during growth (13). The acquisition of bone mass occurs slowly throughout childhood, while it proceeds quickly with the onset of puberty and at the time of growth spurt. Peak bone mass occurs at 12.5 ± 0.90 years in girls and 14.1 ± 0.95 years in boys (13).

The changes of bone sizes and thickness occur more rapidly after epiphyseal fusion and continue until the attainment of peak bone mass during the second decade of life (13). However, the changes of bone shape and bone composition occurring during pubertal development, influence the bone strength (14). Thus, the “bone bank” is built in the first two decades of life, and most of the risk of osteoporosis depends on what occurs in this period. Peak bone mass represents a key determinant of bone health and risk of osteoporosis in adulthood (15). Indeed, it is strictly related to bone strength that in turn is determined by bone mass, bone density, microarchitecture, micro repairs and bone geometry. The attainment of a suitable peak bone mass can prevent fractures both in childhood and adulthood (16–18).

Bone is also a living tissue as old and damaged bone is removed and replaced with newly formed bone. This process of bone renewal and repair is called “bone remodeling”, and it is due to the action of osteoclasts and the osteoblasts (19). In healthy bones, the osteoclast and osteoblast activity are balanced. When this process becomes unbalanced, so that the bone reabsorbing happens faster than bone replacing, bones can become thin and fragile. Bone remodeling is determinant to maintaining the integrity and the mechanical properties of the skeleton (20). This balance is controlled by the RANK/RANKL/osteoprotegerin (OPG) and Wnt/β-catenin pathways which regulate osteoclastogenesis and osteoblastogenesis, respectively (21). Bone remodeling's alterations have been observed in several congenital and acquired pediatric disorders (22). Particularly, in subjects with obesity, the condition of low-grade inflammation activates osteoclasts by up regulating the production of RANKL and other inflammatory cytokines, and inhibiting osteoblastogenesis, thus accelerating bone resorption (23).

## Key determinants of bone health in pediatric age

According to the National Osteoporosis Foundation, bone health is the consequence of both genetic and environmental factors (16). Genetic factors impact skeletal development for approximately 60%–80% (16, 24). Numerous loci linked with low bone mass and osteoporosis have been recognized by Genome Wide Association (GWA) studies (25), although few studies have been conducted in children. Environmental factors, such as diet and physical activity are responsible for 20%–40% of the peak bone mass (16).

### Calcium and vitamin D intake

Adequate calcium and vitamin D intake, in association with physical activity, maximizes peak bone mass and reduces the risk of osteoporosis and fractures in childhood and adulthood (26). The National Institutes of Health provided the recommendations for calcium intake and calcium content of foods (27). The recommended dietary allowance (RDA) for calcium is 1,300 mg for subjects aged 9–18 years (24). Calcium intakes <600 mg/day, may expose to substantial risks of inadequate mineralization (28). Intakes <400 mg/day, especially when combined with low vitamin D levels, represent a risk of rickets and fractures.

A systematic review evaluated the methods and quality of guidelines on calcium and vitamin D supplementation in healthy children (29). The authors observed significant variations on calcium and vitamin D recommendations across 24 guidelines and consensus among countries around the world. The recommended calcium intake for children ranged from 400 to 1,150 mg/day. Additionally, data on vitamin D supplementation at different ages, vitamin D type, and sunlight exposure were conflicting across studies (29).

In a previous study, the supplementation of 800 mg of calcium and 400 IU of vitamin D daily for 6 months resulted in increases in cortical and trabecular bone (30). In contrast, a meta-analysis demonstrated that rising dairy products in the diet significantly improved bone mineral content only in children with low baseline calcium intake (31). Thus, calcium and vitamin D supplementation would appear to have little influence on bone health.

### Physical activity and musculoskeletal health

Muscle and bone interact through both anatomically and mechanically, as well as through paracrine and endocrine signals (32). As regards the mechanical interaction, bones represent a site of attachment for muscles and, in turn, skeletal muscles facilitate locomotion by giving strength to the bone; hence, muscles are the primary source of tension for bones. This is testified by the fact that astronauts experience bone loss and muscle atrophy as they are exposed to an environment that lacks gravity, such as space; as they return to the ground, the speed of recovery that they experience in their muscle mass exceeds that of bone, suggesting muscle contraction is essential for bone recovery. Thus, immobility, aging and other diseases can cause changes in both bone and muscle mass. In addition, skeletal muscles produce factors which regulate bone metabolism. These muscle-secreted factors named “myokines”, include myostatin, interleukin (IL)-6, IL-7, IL-15, IGF-1, FGF-2, irisin, and BAIBA (33).

**Figure 1:**
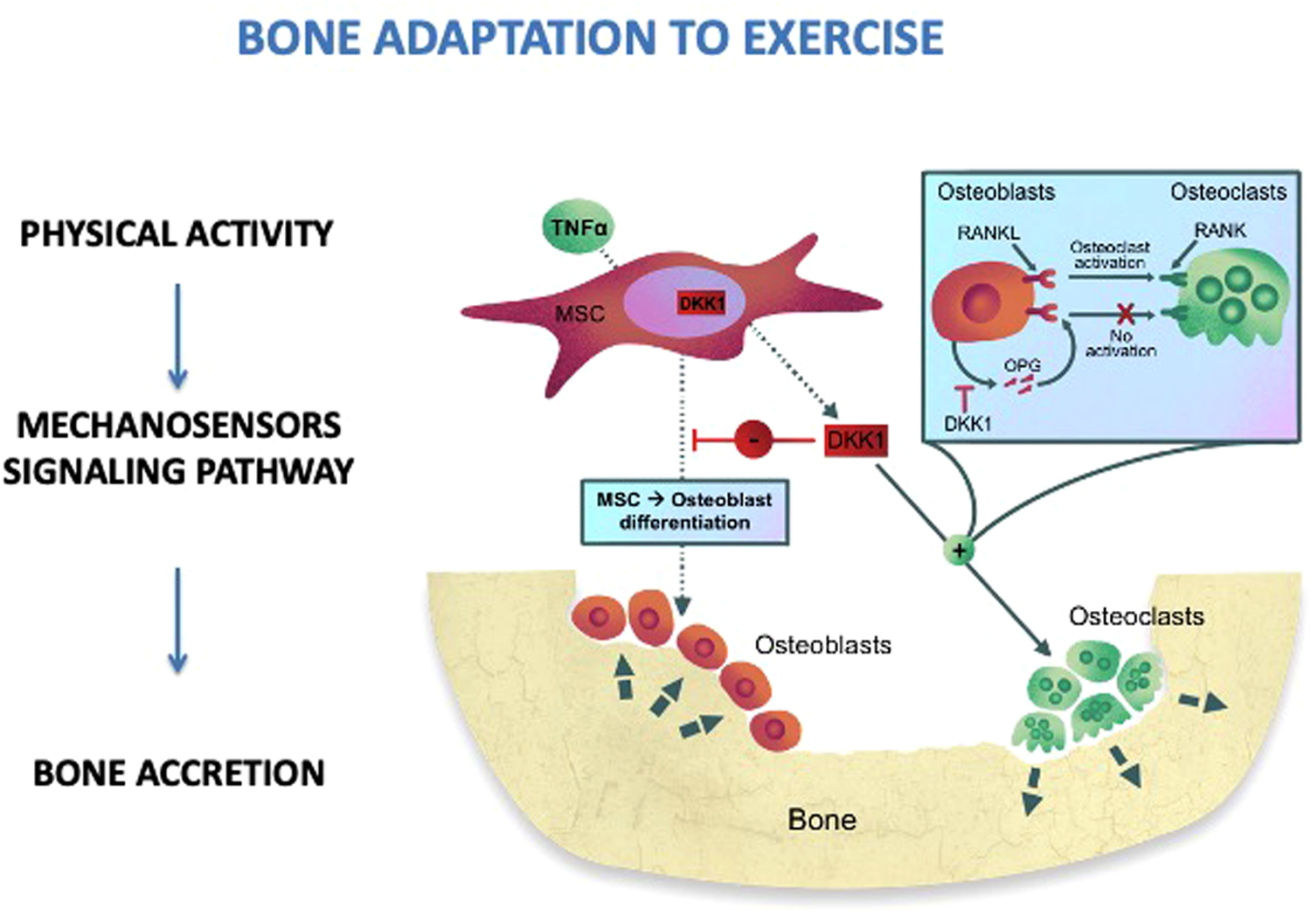
Bone adaptation to exercise. MSC, mesenchimal stem cells; RANKL, receptor activator of nuclear factor kappa-Β ligand; RANK, RANKL/OPG receptor; DKK1, dickkopf-1; OPG, osteoprotegerin; TNFα, tumor necrosis factor alpha.

Physical activity has a positive impact on musculoskeletal health. The skeleton responds to physical stress quickly and bone remodeling starts. Exercise leads to bone adaptation by cellular mechano transduction (34). Quickly, upon exercise, the mechanosensors located through the cells, such as stretch-activated ion channels and integrins, modify their conformation (Figure 1). These conformational changes activate a signaling cascade which determines bone accretion at the site of deformation (34). To obtain an osteogenic response, the bones must undergo such a deformation that exceeds the usual deformation threshold. The threshold varies between individuals and bone sites according to physical activity habits, age, hormone levels, and other metabolic factors (35). Thus, children and adolescents may have a different response to comparable mechanical loads: inactive children may improve bone mass in response to low-impact loads, while active children will need a greater mechanical load (36). The response is regional, allowing specific bone to meet increasing loading requirements. Indeed, a greater bone density has been observed in the dominant arms of baseball, tennis, and squash players with side-to-side variations ranging from 8% to 22% (37). It has also been demonstrated that the magnitude of loading and deviations from normal loading patterns are more important for bone modeling than stimulus duration. Mechanical loading induced by physical activity is necessary to stimulate bone modeling as it provides the stimulus required to develop a robust skeleton (38). Mechanical loads of 3.5 g-force for 3 days/week with 100 loads per session and 7 months of intervention determine an osteogenic effect. Furthermore, the mechanical loading stimulates the Wnt/-catenin signaling pathway, which in turn downregulates osteoclastogenesis and osteoclast activity, thus influencing bone remodeling (39).

Regarding the muscle response to exercise, the increased load contributes to muscle size and strength, primarily as a result of muscle cell hypertrophy rather than hyperplasia (40). The increase in muscle mass results in an inhibition of myostatin which hence regulates muscle mass. Nonetheless, it is not yet clear why only the muscles under load increases muscle mass, while the decrease in circulating levels of myostatin resulting from exercise should affect the whole body (41). This suggests that other unknown soluble factors produced by skeletal muscles after exercise may contribute to increases in muscle size and strength. On the other hand, during aging and immobility, the physiological changes caused by skeletal unloading determine the onset of sarcopenia, characterized by a decrease in the size (atrophy) and number (hypoplasia) of muscle fibers. Therapeutic strategies to treat sarcopenia are currently aimed at targeting the myostatin/activin signaling mechanism, on the basis of the principle of “muscle-bone crosstalk”.

### Irisin: a myokine involved in the crosstalk between muscle and bone

Irisin is a myokine firstly identified for its role in inducing browning of white adipose tissue and increasing energy expenditure (42). Then, both in human and mouse it has been demonstrated that irisin is also involved in glucose homeostasis by promoting liver glycogen synthesis and inhibiting gluconeogenesis (43, 44). Further role of this myokine in cognitive functions, learning, and memory has been recognized (45). In humans irisin represents a link between physical activity and metabolic homeostasis, as it mediates the beneficial effects of exercise on glucose and lipid metabolism, and it provides to maintain musculoskeletal homeostasis (46, 47). This evidence strengthens the hypothesis that muscle can be considered an endocrine organ. Studies of proteomics and transcriptomics identified the composition of the skeletal muscle “secretome” leading to identification of several myokines (48). Most of the proteins identified in cultures of myotube were predicted as putative secreted proteins, pointing out that skeletal muscle acts as an endocrine organ. Functional analysis suggests their role in skeletal muscle as paracrine regulators of oxidation, hypertrophy, angiogenesis, and extracellular matrix (49). These myokines are also involved in the regulation of body weight, inflammation, insulin sensitivity, and cognitive function. Thus, muscle derived regulatory RNAs could represent a novel frontier for the treatment of chronic diseases. Exercise as downhill running, eccentric exercise, and resistance training determine a systemic marked cytokine response, with a higher degree of muscle involvement (50). However, a strong elevation of several cytokines and chemokines in skeletal muscle has been described after exercise with long duration or high intensity (51), while a less evident or absent response has been observed after moderate intense physical activity (52). The direct involvement of irisin in bone metabolism, by inducing the differentiation of bone marrow stromal cells into mature osteoblasts, has been demonstrated (53). In healthy children, irisin serum levels positively correlate with bone mineral status and negatively with inhibitors of Wnt signaling pathway (54). Furthermore, high irisin levels correlate with a better glycemic control and bone health in children affected with type 1 diabetes (55). Zhang et al., confirmed the positive regulatory effect of irisin on bone during physical exercise, as they found an increased expression of biochemical markers of bone formation and irisin increase in bone tissue after 2 weeks of free wheel-running exercise (56). Exercise-induced irisin, or exogenous administration of irisin, can prevent bone loss, as demonstrated in hind-limb suspended mice (47). However, the true effect of physical activity in promoting secretion of irisin is still debated. Some studies reported a huge effect of exercise in promoting irisin release (45–47), while others did not document any changes in irisin levels after both acute and chronic exercise (57, 58). Probably, this disagreement depends on the type and duration of exercise, and the type of assay for irisin assessment. Conversely, serum irisin levels has been identified as a predictive biomarker for sarcopenia (59, 60). Moreover, irisin can activate the differentiation of osteoblasts under microgravity conditions by promoting the secretion of β-catenin protein and increase OPG levels (61).

Furthermore, body composition is also a relevant factor for musculoskeletal health, in fact age-related loss of skeletal muscle (sarcopenia) is often associated with obesity (“sarcopenic obesity”). Increased myostatin levels have been shown to result in low levels of muscle tissue mass and at the same time inhibit insulin signaling, muscle mitochondrial biogenesis, lipid oxidation and energy expenditure, therefore this myokine could play a key role in sarcopenic obesity (62).

## The impact of exercise interventions on bone strength

Physical exercise and healthy eating habits during growing increases the probabilities of accruing bone, and potentially delays osteoporosis in adulthood. Literature data on the effectiveness of physical activity interventions in childhood and adolescence are heterogeneous.

A meta-analysis of 27 studies found a significant effect of weight bearing activity on BMC and BMD (63). Children involved in school-based exercise programs for 9 months showed higher whole-body, femoral neck and total hip BMC compared with their counterparts not involved in exercising (64). Furthermore, a persistence of the benefits 3 years after ceasing the intervention has been observed. Longitudinal studies reached different conclusions regarding whether the osteogenic effect depends on continuing physical activity into adulthood (65–67). Subject who practiced sports during childhood showed a greater hip BMC in adulthood than their sedentary counterparts (68). A cross-sectional cohort study investigated the longstanding effects of soccer on BMD and fracture risk, demonstrating a higher BMD, and lower risk of fractures in the athletes 30 years after retirement (69).

A recent study demonstrated that the age at which children first start walking might affect their bone strength in later life (70). Ireland et al., examined the association between walking age and bone mineralization by Dual-energy x-ray absorptiometry (DXA) and Quantitative Computed Tomography (pQCT) in subjects aged between the ages of 60 and 64 years (70). Later independent walking age has been associated with lower BMC, suggesting that early mechanical loading on the skeleton might influence bone strength development. A systematic review of the literature evaluated the changes induced on bone mineralization by ball games, dancing, jumping and other physical activities. The results of this study showed that weight-bearing exercise in childhood had a positive effect on bone strength, while exercise performed during prepubertal and peripubertal age caused an increase in bone mineral accrual (71). These results have been confirmed by other studies which observed that, although osteogenesis and bone anabolism are more pronounced during the peripubertal phase, the period immediately preceding puberty represents a “window of opportunity” in which the skeleton is more sensitive to mechanical stress (35).

### The impact of physical activity on musculoskeletal health

In this section we reported the most important clinical trial conducted in the last 5 years that investigated weight-bearing physical activities improving musculoskeletal health. Table 1 shows the recent data on the effects of sport interventions on bone mineralization.

**Table 1:** Clinical trial investigated weight-bearing physical activities improving musculoskeletal health.

Study	Participants	Exercise intervention details	Bone mineralization outcomes
Vlachopoulos et al., 2017 (64)	116 males aged 13.1 0.1 years evaluated at baseline and at 12-month (follow up)	37 swimmers 37 footballers 28 cyclists 14 controls Duration: more than 3 h per week	- Footballers had higher improvement in BMC compared to cyclists and swimmers - No significant difference between swimmers and cyclists was found
Larsen et al., 2016 (68)	295 Danish school children aged 8–10 years	96 small-sided ball game group (SSG) 83 strength training group (CST) - 116 controls Duration: 3 × 40 min/week over 10 months	- Both training types resulted in higher change scores in postural balance - SSG group had higher change scores in leg aBMD compared with CST and controls
Bielemann et al., 2019 (69)	4,106 adolescents from the 1993 Pelotas Birth Cohort Physical activity assessed at 11, 15, and 18 years of age by self-report and at 18 years by accelerometry	Two groups: - 150 min/week of moderate physical activity (MPA) - at least 75 min/week of vigorous physical activity (VPA)	- Time spent in MPA at 11 and 15 years was not associated with aBMD improvement - VPA at all time points was positively related to aBMD improvement in boys - VPA was related to higher a BMD at 18 years of age in girls
Zribi et al., 2022 (74)	39 adolescents aged 11 ± 1 years at baseline and at 12-month (follow up)	20 prepubescent boys, volleyball players 19 controls Duration: for volleyball players 4–6 h of training plus one competition game a week for at least 18 months in addition to 2 weekly physical education sessions at school (of 50 min each) Controls: physical education session at school (50 min each)	At follow-up, volleyball players gained more BMD in whole body than controls; a close correlation was observed between the increment of whole body lean mass and increased BMD and BMC in whole body

The PRO-BONE study explored the effect of 12-months involvement in osteogenic (football) and non-osteogenic (swimming and cycling) sports in 116 adolescent male athletes aged 13.1 years ±1.0 (72). The authors observed that after 12-months participation, footballers showed significantly higher BMC at most skeletal sites than swimmers and cyclists. A previous study demonstrated that 8 months of sport-specific training improved total body BMD by 2.9% in footballers, but not in swimmers (73). These results confirm previous studies that reported that swimming and cycling have no effect on bone strength, possibly due to the low ground reaction forces produced during exercise (74, 75). Therefore, football improves bone strength compared to non-osteogenic sports which should be combined with weight-bearing activities.

The randomized controlled trial FIT FIRST involving 295 Danish children aged between 8 and 10 years evaluated the effects on bone mineralization and muscular structure after 10 months of high-intensity school training (3 × 40 min/week) consisting of team soccer and other ball games or circuit training with weight-bearing exercises (76). The authors observed that both high-impact school interventions improved musculoskeletal health in children. The observations confirmed that in childhood the skeleton adapts to the physiological changes induced by physical training and suggested that both high-intensity interval program and odd-impact training may improve musculoskeletal health.

Bieleman et al., examined the relations between physical activity and areal BMD (aBMD) according to intensity of exercise in several sports: outdoor soccer, indoor football, athletics, basketball, volleyball, tennis, handball, dance, gymnastics, martial arts, swimming, trapper, and playing bat (77). Vigorous physical activity improved aBMD more than moderate physical activity, especially in boys. In particular, the involvement in vigorous activities from the middle to the end of adolescence seems to be related to higher aBMD. Other studies described differences induced by physical activities on aBMD between boys and girls (78–80). The better impact of vigorous-intensity physical activity on bone density in boys than in girls may be explained by the increased sensitivity to mechanical loading in boys during adolescence and exposure to testosterone which increases bone and muscle mass (81).

Zribi et al. evaluated longitudinally the consequences of 1-year of volleyball on BMD and BMC, assessed by DXA (82). Volleyball is a team sport which includes different movements such as accelerations and decelerations, rapid changes of direction and repetitive jumps. All actions generate high stresses on the upper and lower limbs from the reaction forces produced by the jumps, which measure three to six times the body weight.

This study demonstrated that playing volleyball 4–6 h per week for approximately 1 year resulted in higher BMD and BMC at all skeletal sites analyzed in prepubertal boys compared with non-physically active controls. In volleyball the mechanical forces acting on the bones derive both by the high reaction forces produced by the impact with the ground in the jump, and by the muscular contractions which pull their bone attachment.

## Physical activity in rare diseases

Physical activity has a key role in the care of patients affected with rare diseases, as it exerts both physic and psychological effects, pointing to increase quality of life. In addition, there is evidence that physical activity can improve bone health in some skeletal and extra-skeletal rare diseases.

Rheumatic and musculoskeletal diseases include a group of systemic diseases such as osteoarthritis, rheumatoid arthritis, systemic lupus erythematosus, axial spondyloarthritis, psoriatic arthritis, systemic sclerosis, characterized by pain, disability, and low quality of life. Patients with rheumatic and musculoskeletal disorders experience loss of mobility, loss of autonomy, and higher mortality rates. Consequently, these disorders have a high impact on the social and health system.

The recent recommendations of the European League Against Rheumatism (EULAR) taskforce suggested that exercise interventions improve pain and functions, although the size of the effect varied by type of diseases and type of intervention (83). As weight gain has been associated with worse outcomes for most of these conditions, maintaining an adequate body weight is also recommended.

Physical activity is essential for children with hemophilia, a rare X-linked bleeding disorder caused by a missing or defective clotting factor, to maintain joint movement, reduce joint bleeding, develop muscle mass and strength, and prevent secondary chronic disease and osteopenia/osteoporosis (84–86). In the past, since there was no treatment for hemophilia, the affected children were forbidden to exercise because the risk of bleeding. Current therapies not only treat acute bleeding, but also prevent it. It is sufficient to pre-administer the deficient clotting factor and the risk of bleeding is temporarily reduced. The availability of effective and safe drugs drives patients and caregivers to insistently ask doctors to start physical activity as for their peers (87).

Although for a hemophilic child under pharmacological treatment there are no contraindications for practicing sports, the sport promotion for hemophilic children is yet an obstacle course (88).

Different guidelines indicated hemophilic subjects' sports participation according with type and severity (89). In addition, the selection of activities should consider individual preferences, abilities, and physical conditions. However, high-impact sports such as rugby, boxing, soccer and basketball, or sports with a higher risk of injury are often discouraged despite good prophylaxis. The National Hemophilia Foundation (NHF) has suggested guidelines for athletic participation of patients with bleeding disorders (90). For hemophilic patients, a minimum of 60 min of exercise per day, with adequate supervision, is recommended after receiving prophylaxis.

To overcome the problem and allow physical activity for every child with hemophilia, the Italian study group has drawn up some recommendations which invite gradual training before accessing intense physical activity. Gradual training allows the individual subject to understand which and what physical activity practice and favors the possibility of daily moderate physical activity to limit the chronic evolution of arthropathy (91).

Prader-Willi syndrome (PWS) is the most frequent form of genetic obesity. It is characterized by severe hypotonia and feeding problems in early infancy, followed by excessive eating and gradual development of severe obesity (92). In adulthood, PWS patients develop cardio-respiratory diseases, psychiatric disorders as well as various comorbidities such as muscle weakness and scoliosis. Encouraging physical activity is an essential objective of the management of PWS both in children and adults. In the last years, several studies have considered physical activity in PWS patients (93–95). Although most studies have described a low exercise in patients with PWS, it is not clear whether the decreased physical activity in these patients is related to obesity *per se*, or to the physical and intellectual disabilities related to this syndrome. A recent systematic review which considered controlled trials, single-group interventions, observational, and qualitative studies reported that only 5%–8% of PWS children (93, 96, 97) and 15%–25% of adults (94, 98) met the WHO physical activity and sedentary behavior guidelines (99). According with WHO guidelines, 60 min/day of moderate-to-vigorous physical activity is suggested for children, and 150–300 min/week (i.e., at least 30 min/day) for adults (98). Replacing sedentary lifestyle with physical activity has beneficial effects for lifelong health preservation, particularly for individuals with low physical activity levels such as PWS patients (100).

Recently, a protocol for a randomized trial on increasing resistance training in young people with PWS has been started (101). The purpose of this study is to establish whether progressive resistance training is effective in improving muscle strength in PWS subjects, understand participants' experiences and identify factors influencing implementation, and determine long-term efficacy in terms of healthcare expenditure.

## Conclusion and future directions

The skeleton is a structure made up of living tissue that grows, repairs, and renews itself. It plays a supportive role for muscles and a protecting role for internal organs. Furthermore, the skeleton influences energy metabolism through a continuous interaction with cytokines derived from both adipose and muscle tissue, and with insulin. There is substantial evidence that skeletal muscle secretes factors which act as mediators of endocrine signaling and are also implicated in the favorable effects of exercise. Skeletal muscle “secretoma” during exercise has not yet been described, but research has developing modified myokines with the aim of supporting the treatment of chronic diseases.

Adequate nutrition and physical activity can influence musculoskeletal health; however how exercise during pediatric age affects bone health remains to be investigated. Previous studies have demonstrated a cumulative effect of vigorous physical activity during adolescence on BMD, suggesting that any time in adolescence is a window of opportunity to increase bone mass. The mechanical forces acting on the loaded bones are generated both by the high reaction forces produced by the impact with the ground in sports such as high jump, basketball, and volleyball, and by the muscle contractions that pull their attachment on the skeleton. This agrees with previous studies that have shown that lean mass development is the best predictor of bone mass accumulation. Osteogenic sports, such as football, result in a higher BMC than non-osteogenic sports, such as swimming and cycling, in which physical activities should be combined with weight-bearing movements to optimize bone growth.

Studies of larger cohorts of children and adolescents addressing issues such as the complex interaction between bone, gut, white and brown adipose tissue, nutrition, and physical activity will be needed in the future to provide new insights into this fascinating field of metabolic endocrinology.
